# Assessing linkage to and retention in care among HIV patients in Uganda and identifying opportunities for health systems strengthening: a descriptive study

**DOI:** 10.1186/s12879-018-3042-8

**Published:** 2018-03-23

**Authors:** Caroline E. Boeke, Vennie Nabitaka, Andrea Rowan, Katherine Guerra, Arnold Kabbale, Barbara Asire, Eleanor Magongo, Pamela Nawaggi, Vivienne Mulema, Betty Mirembe, Victor Bigira, Andrew Musoke, Cordelia Katureebe

**Affiliations:** 1 Clinton Health Access Initiative (CHAI), Boston, USA; 2 CHAI, Kampala, Uganda; 3 Ministry of Health, Kampala, Uganda; 4 383 Dorchester Road, Suite 400, Boston, Massachusetts 02127 USA

**Keywords:** HIV, Africa, Service delivery, Linkage to care, Retention in care

## Abstract

**Background:**

While antiretroviral therapy (ART) availability for HIV patients has increased dramatically in Uganda, patient linkage to and retention in care remains a challenge. We assessed patterns of engagement in care in 20 Ugandan health facilities with low retention based on national reporting.

**Methods:**

We assessed patient linkage to care (defined as registering for pre-ART or ART care at the facility within 1 month of HIV diagnosis) and 6-month retention in care (having a visit 3-6 months after ART initiation) and associations with patient−/facility-level factors using multivariate logistic regression.

**Results:**

Among 928 newly HIV-diagnosed patients, only 53.0% linked to care within 1 month. Of these, 83.7% linked within 1 week. Among 678 newly initiated ART patients, 14.5% never returned for a follow-up visit at the facility. Retention was 71.7% according to our primary definition but much lower if stricter definitions were used. Most patients were already falling behind appointment schedules at their first ART follow-up (median: 28 days post-initiation vs. recommended 14 days). 27.3% of newly-initiated patients had follow-up appointments scheduled 45+ days apart rather than monthly per national guidelines. Linkage and retention were not strongly correlated with each other within facilities (r_s_ = 0.06; *p* = 0.82). Females, adolescents, and patients in rural settings tended to have lower linkage and retention in multivariable-adjusted models.

**Conclusions:**

Linkage support may be most critical immediately after testing positive, as patients are less likely to link over time. More information is needed on reasons for appointment schedules by clinicians and implications on retention.

**Trial registration:**

This study was registered in the Pan African Clinical Trial Registry database (#PACTR201611001756166).

**Electronic supplementary material:**

The online version of this article (10.1186/s12879-018-3042-8) contains supplementary material, which is available to authorized users.

## Background

Antiretroviral therapy (ART) coverage for HIV patients has been increasing steadily in Uganda. Of the estimated 1.5 million people living with HIV in Uganda in 2015, [1] approximately half were on ART [2]. Coverage has increased due to targeted testing in high-risk populations, improving linkage to care systems, Option B+ programs (2013 onwards) and treatment of all pediatric patients (2014 onwards). In January 2017, the Uganda Ministry of Health (MOH) began to scale up a national Test and Start program to provide ART for all patients regardless of CD4 count or clinical stage. Despite these tremendous gains in treatment accessibility and scale-up, linkage to care among patients who test positive and retention in care among patients on treatment remains a challenge. Based on recent government data, national 12-month retention after ART initiation for all populations is 75.5%, with some geographic variation, [3] and other recent studies in Uganda have reported retention ranging from ~ 75 to ~ 90% in pediatric patients and adults [4–6]. However, some facilities report substantially lower retention than average. The percentage of patients in the general population who link to care after testing positive has not been well described, although studies in key populations have cited very low linkage [7–10].

High attrition rates pose a serious concern to HIV programs as treatment discontinuation leads to increased viral load and associated increases in morbidity, mortality, incidence of drug resistance and risk of transmission, ultimately undermining the benefits achieved from implementing treatment programs. Additionally, high attrition will require costly efforts to re-find and re-initiate patients to meet and sustain Uganda’s ART scale-up targets. Thus, there is an increasing need for focused attention on linkage to and retention in care to improve patient outcomes and successfully control Uganda’s HIV epidemic.

A number of groups have studied barriers to linkage to and retention in ART care in Uganda and neighboring countries. Barriers reported by patients included transportation costs, long wait times, and stigma [11]. In one Ugandan study, poverty, unemployment, and residence in rural areas were associated with greater loss to follow-up in a pre-ART program [12]. Several studies have observed poorer retention among the sickest patients (as defined by CD4 count, staging, opportunistic infections, and other factors), perhaps suggesting that some patients who were considered lost to follow-up (LTFU) had actually died or were too sick to travel to facilities for care [4, 13, 14]. However, limited information is available on the facility-level factors that predict linkage and retention.

More evidence is needed on the patterns around and predictors of patient engagement in care in order to generate evidence on scalable and sustainable interventions for improvement. We retrospectively assessed patient linkage to and retention in care and service delivery barriers among 20 health facilities in Central Uganda. While this group of facilities is not nationally representative, the findings from this analysis can help to identify patterns in poorly performing facilities and opportunities for health systems strengthening.

## Methods

### Study design

Twenty facilities with low retention were randomly selected across 14 districts in Central Uganda (region chosen due to logistical feasibility) to participate in a study involving an intervention to promote proactive follow-up and counseling practices. Facility eligibility criteria included offering ART to pediatric and adult patients starting January 1, 2015 or earlier, demonstrating a low estimated annual retention among patients on ART (35-75% in 2015 in calculations using national District Health Information System (DHIS) 2 estimates), and having a high ART patient volume (> 120 patients enrolled in 2015 according to DHIS 2). All eligible facilities were level III or IV, medium-sized health facilities offering general services but no specialized services. Eligible facilities located on islands (*n* = 4) were excluded for logistical reasons and one specialized mental health center was excluded.

Data were retrospectively collected on the previous 9 months using standard testing and treatment registers and patient care cards at each facility. We also collected data from ART in-charges on current practices at each facility to counsel patients and follow up/track patients who missed appointments as well as challenges with the current patient follow-up and counseling systems. Data were collected electronically by professional data collectors and senior study staff using SurveyCTO software (2016 Dobility, Inc., Cambridge, Massachusetts) on tablets.

### Linkage to care

Patients of all ages who tested HIV-positive at study facilities between December 25, 2015 and June 25, 2016 were sampled for inclusion in the linkage assessment. Based on preliminary power calculations, data collectors systematically sampled HIV-positive patients in the HIV Testing and Counselling Register such that a minimum of 40 patients were sampled per facility and the smallest possible number of patients beyond that were sampled. However, all pediatric patients were sampled. Also, if the number of patients testing during the relevant time period was less than 60 patients, all patients were sampled. Pre-ART and ART registers were assessed in the 3 months after diagnosis to confirm which HIV-positive patients linked to care at the facility. Due to logistical and budgetary constraints, it was not feasible to follow up at other facilities to search for patients who did not link to care at the facility where the test was conducted.

The primary definition of linkage to care was defined as registering for pre-ART or ART care at the facility within 1 month (30 days) of HIV diagnosis, as the WHO recommends timely linkage to care to ensure that patients access appropriate treatment as soon as possible [15, 16]. Patients who self-reported transferring to another facility for care or reportedly died during follow-up were excluded from the measure of linkage, as were patients with unknown dates of linkage. We also assessed linkage within 3 months (90 days) of diagnosis to match the definition used in previous studies [7, 17, 18] as well as shorter time windows.

### Retention in care

All patients (of all ages) who initiated ART at study facilities between December 25, 2015 and March 25, 2016 and were listed on the ART register were included in the retention assessment. Data on patient demographics and scheduled and attended ART appointments through 6 months post-initiation were extracted from patient care cards.

Our a priori primary definition of 6-month retention was attending at least 6 ART appointments in the first 6 months after ART initiation. This represents full adherence to the ART visit schedule in the National ART Guidelines as of 2016, in which patients should attend an ART appointment 2 weeks after ART initiation and monthly thereafter for the first year after initiation. Revised guidelines in 2017 required appointments at 2 weeks, monthly for 3 months, and quarterly thereafter. We also assessed six-month retention according to the Uganda MOH definition: Having a visit within the previous quarter (3-6 months after ART initiation). To understand patient appointment spacing, we calculated median number of patient appointments over 6 months and compared appointment spacing to national guidelines.

This analysis is from the baseline assessment and includes pre-intervention data from December 2015-September 2016.

### Statistical analysis

Proportions and medians/interquartile ranges (IQR) were calculated to describe program and patient characteristics. Predictors of linkage and retention were assessed using logistic regression models using cluster-robust standard errors given facility-level data clustering. The missing indicator method was used to account for missing data [19]. Variables that were statistically significant or borderline significant in univariate analyses were included in the multivariable model. The correlation between linkage and retention at facilities was calculated using a Spearman’s correlation coefficient. Analyses were completed in StataSE 13 (College Station, Texas) and Microsoft Excel 2011 (Seattle, Washington).

## Results

Detailed description of the patient follow-up practices at study facilities and challenges identified can be found in Supplemental Digital Content (Additional file 1).

### Linkage to care

**Fig. 1:**
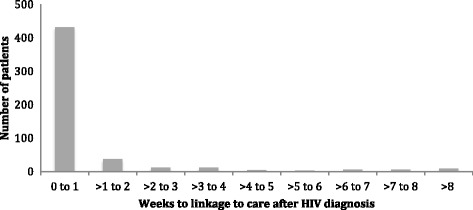
Time to linkage to pre-ART or ART care (weeks) among the 516 patients who linked to care within 3 months of HIV diagnosis

Linkage was assessed among 928 newly diagnosed HIV patients across the 20 study facilities (Table 1). Of the 949 patients in the dataset, 20 HIV-positive patients self-reported transferring to another facility for care and 1 reportedly died following diagnosis; these patients were excluded from the primary analysis. The median (IQR) age of the patients was 29 (21-38) years. 60.0% of patients were female. Among patients who linked to care with known stage, 6.2% were stage III or IV at diagnosis. 29.1% of patients linked to care on the day of HIV diagnosis, 53.0% linked within 1 month, and 55.6% linked within 3 months. Figure 1 shows time to linkage to care and Additional file 2: Table S1 shows alternative definitions of linkage to and retention in care. The median (IQR) time to linkage was 0 (0-6) days. Among patients who linked within 3 months of diagnosis, most (83.7%) linked within 1 week.

**Table 1:** Patient and facility characteristics in relation to linkage to care^a^

Characteristic	N	N (%) linked to care within 1 month	Univariate OR (95% CI)	*p*-value	Multivariate OR (95% CI)	*p*-value
Sex						
Female	557	50.4%	0.77 (0.58-1.03)	0.08	0.80 (0.60-1.06)	0.12
Male	371	56.9%	Ref		Ref	
Age group						
< 10 years	73	57.5%	1.21 (0.81-1.82)	0.35	1.10 (0.73-1.67)	0.65
10-18 years	78	39.7%	0.59 (0.36-0.96)	0.03	0.58 (0.35-0.96)	0.03
19-48 years	688	53.5%	Ref		Ref	
49+ years	74	64.9%	1.65 (0.95-2.86)	0.07	1.61 (0.94-2.75)	0.08
Missing	15	66.7%	–		–	
Clinical stage^b^						
I	290	–	–		–	
II	90	–	–		–	
III or IV	25	–	–		–	
Missing	523	–	–		–	
Facility location						
Rural/remote (*n* = 14)	616	49.7%	0.67 (0.46-0.98)	0.04	0.64 (0.43-0.95)	0.03
Semi−/peri-urban (*n* = 6)	312	59.6%	Ref		Ref	
Facility size of expert client staff pool						
1 or fewer (*n* = 3)	98	52.0%	0.88 (0.34-2.27)	0.79	–	
2-4 (*n* = 9)	445	51.2%	0.85 (0.57-1.25)	0.41	–	
5+ (*n* = 8)	385	55.3%	Ref		–	
Facility level						
III (*n* = 9)	377	52.3%	0.95 (0.59-1.52)	0.83	–	
IV (*n* = 11)	551	53.5%	Ref		–	
Days ART offered per week						
1 (*n* = 9)	384	47.9%	0.83 (0.53-1.30)	0.41	–	
2-3 (*n* = 8)	394	58.1%	1.25 (0.84-1.85)	0.28	–	
4-5 (*n* = 3)	150	52.7%	Ref		–	

^a^Linkage to care measured within 1 month among 928 patients newly diagnosed with HIV at 20 facilities in Uganda. Logistic regression models used cluster-robust standard errors to account for clustering by health facility. Variables that were statistically significant or borderline significant in univariate models were included in the multivariable models

^b^Clinical stage data was only available for patients who linked to care; therefore, it was not possible to include this information in the models

In univariate models, linkage was slightly lower in women than men, although the difference was not statistically significant (50.4% vs. 56.9%; *p* = 0.08) and was poorest in adolescents (ages 10-18 years) compared to adults (ages 19-48 years) (39.7% vs. 53.5%; *p* = 0.03). In particular, adolescent females had the lowest linkage (35.5%) of any sex/age category combination. In addition, in univariate models, significantly lower linkage to care was seen in rural/remote facilities compared to semi−/peri-urban facilities (49.7% vs. 59.6%, *p* = 0.04). No differences were seen by facility level, the number of days per week that ART was offered at the facility, or the number of expert clients at the facility. We also assessed other facility-level characteristics such as year of start of implementation of ART program, and reports of stockouts, but these variables were not associated with linkage to care. In multivariable models, significantly lower linkage was seen in adolescents and in patients in rural settings.

### Retention in care

**Fig. 2:**
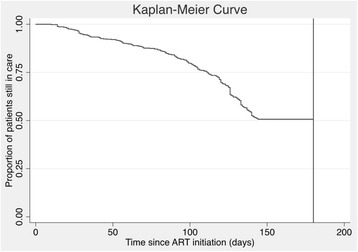
Kaplan-Meier curves showing the proportion of patients in care over time. Patients with a visit in the final 35 days of follow-up were considered to be in care at 180 days

Six-month retention was calculated among 678 patients newly initiated on ART (Table 2). Twenty-four patients were excluded from the original dataset of 713 patients because they self-reported transferring to another facility and 11 were excluded because they reportedly died over the 6 months of follow-up. The median (IQR) age among newly ART-initiated patients was 30 (24-39) years. 60.2% of patients were female. 14.5% of patients never returned for a first follow-up appointment after initiation. The median (IQR) number of appointments for patients over 6 months was 4 (2-5). 71.7% of patients were retained at 6 months according to the MOH definition of at least one visit in months 3-6. However, when using a more intensive definition (and our primary definition of retention a priori) of at least six visits by 6 months, only 6.9% of patients adhered to this appointment schedule. Because this value was so low, the MOH definition of retention was used for follow-up analyses. A Kaplan-Meier curve with the proportion of patients in care is shown in Fig. 2; LTFU appeared to accelerate slightly over time.

**Table 2:** Patient and facility characteristics in relation to retention in care^a^

Characteristic	N	N (%) retained in care	Univariate OR (95% CI)	*p*-value	Multivariate OR (95% CI)	*p*-value
Sex						
Female	408	69.4%	0.75 (0.58-0.97)	0.03	0.76 (0.60-0.97)	0.03
Male	270	75.2%	Ref		Ref	
Age group						
< 10 years	30	73.3%	1.08 (0.41-2.84)	0.87	1.12 (0.41-3.03)	0.83
10-18 years	20	50.0%	0.39 (0.16-0.99)	0.05	0.43 (0.17-1.06)	0.07
19-48 years	541	71.7%	Ref		Ref	
49+ years	64	79.7%	1.55 (0.79-3.04)	0.21	1.46 (0.75-2.85)	0.26
Missing	23	65.2%	0.74 (0.47-1.16)	0.19	0.83 (0.46-1.49)	0.53
Facility location						
Rural/remote (*n* = 14)	389	67.6%	0.62 (0.41-0.93)	0.02	0.62 (0.39-0.98)	0.04
Semi−/peri-urban (*n* = 6)	289	77.2%	Ref		Ref	
Facility size of expert client staff pool						
1 or fewer (*n* = 3)	25	64.0%	0.62 (0.41-0.94)	0.03	0.82 (0.52-1.28)	0.37
2-4 (*n* = 9)	328	69.8%	0.81 (0.49-1.33)	0.40	0.86 (0.56-1.33)	0.50
5+ (*n* = 8)	325	74.2%	Ref		Ref	
Facility level						
III (*n* = 9)	189	70.9%	0.95 (0.63-1.42)	0.80	–	
IV (*n* = 11)	489	72.0%	Ref		–	
Days ART offered per week						
1 (*n* = 9)	185	67.6%	0.72 (0.48-1.08)	0.11	–	
2-3 (*n* = 8)	380	72.9%	0.93 (0.54-1.60)	0.79	–	
4-5 (*n* = 3)	113	74.3%	Ref		–	

^a^Retention in care measured at 6 months among 678 patients newly initiated on ART at 20 facilities in Uganda. Logistic regression models used cluster-robust standard errors to account for clustering by health facility. Variables that were statistically significant in univariate models were included in the multivariable models

In univariate models, retention was slightly lower in women compared to men (69.4% vs. 75.2%; *p* = 0.03) and was very low in adolescents versus adults 19-48 years (50.0% vs. 71.7%; *p* = 0.047). Lower retention was observed in rural/remote environments compared to semi−/peri-urban (67.6% vs. 77.2%, *p* = 0.02), and in facilities with fewer expert clients to provide counseling and follow up with patients who missed appointments (64.0% among facilities with 1 or fewer expert clients vs. 74.2% among facilities with 5+ expert clients; p = 0.03). Facilities offering ART appointments on fewer days (i.e., once per week compared to 4-5 times) appeared to have slightly lower retention, although this was not statistically significant. No differences in patient retention were seen by facility level, date of ART program initiation at the facility, or other facility-level characteristics. When all statistically significant characteristics were included in a multivariable model, only female sex and rural location remained statistically significant predictors of low retention.

### Linkage in relation to retention

There was substantial variability in linkage and retention by facility and district. Linkage and retention were not strongly correlated with each other within facilities (r_s_ = 0.06; *p* = 0.82). The interclass correlation coefficient (ICC/rho) was 0.047 for linkage and 0.025 for retention. Ninety-eight patients who were found to link to care in the linkage dataset also appeared in the retention dataset. Of those, patients who linked to care within the first week of HIV diagnosis appeared to be more likely to be retained in care compared to patients who linked after 1 week, although with limited statistical power in this analysis, the difference was not statistically significant (68.7% vs. 53.3%; *p* = 0.18).

### ART appointment spacing

The median (IQR) time between ART initiation and first follow-up appointment was 28 days (27-42) per person, compared to 14 days as per national guidelines. 27.3% of newly initiated patients had follow-up appointments scheduled at least 45 days apart. This meant that the median (IQR) time between subsequent appointments was 39 (28-56) days per person, versus 28 days as per national guidelines.

## Discussion

Linkage and retention were low (53.0 and 71.7%, respectively) at the 20 study facilities in Central Uganda and tended to be lower in women, adolescents, and patients living in rural settings. Lower retention was seen in facilities with 1 or fewer expert clients to follow up with patients who missed appointments compared to 5 or more expert clients, although this association did not remain statistically significant in multivariable models. Of note, most patients who tested positive for HIV and subsequently linked to care did so within 1 week of diagnosis, indicating that linkage support may be most critical in the first week after testing positive. There was substantial variability in linkage and retention between facilities, and linkage was not strongly correlated with retention. Numerous facility-level factors were identified that may have contributed to low linkage and retention, such as poor recordkeeping systems, inadequate funding for patient phone follow-up and home visits, and limited staff. In addition, poor staff supervision and motivation appeared to be an issue at some facilities. Finally, we observed that a substantial proportion of patients appeared to be receiving multi-month appointments in the first months after ART initiation, contrary to current national guidelines.

The low linkage and retention at facilities in this study were not surprising given that these facilities were selected based on suspected poor patient retention based on national reporting and justify increased focus on strengthening health systems at facilities like these. Facility-level inefficiencies and constraints identified by facility staff such as limited funding and staff capacity for phone follow-up and home visits to patients who miss appointments, lack of private space for patient counseling, and stockouts echo common barriers to care in sub-Saharan Africa that have been previously described in the literature [20–22]. Factors that were found to be significantly associated with lower linkage and retention in this study have been noted previously. Adolescents are known to be a difficult to reach population with high attrition [23] and deserve greater focus in future interventions to reduce attrition. Overall, a greater number of women are engaged in HIV care, [24, 25] consistent with the fact that substantially more of the patients in these datasets were women. The biggest gender gap appears to be due to fewer men than women accessing HIV testing services [26]; most women are diagnosed during routine prenatal testing and thus represent a broader population that may have a similar or slightly lower likelihood of remaining in care once testing positive compared to men, depending on the context. Patients in rural areas may require longer transportation times to get to facilities which could explain why lower linkage and retention were seen in these settings; access to HIV services tends to be lower in rural populations [27, 28]. Finally, several of the rural/remote study facilities serve highly mobile patient populations such as fisherfolk living on Lake Victoria and migratory cattle farmers. HIV prevalence among Ugandan fisherfolk is high relative to the general population (15-40%) [29, 30] and fisherfolk are known to have poor linkage (e.g., 38% within 3 months of outreach event-based testing) [9]. Barriers to care in this population include limited healthcare access, quality of care, and social support, high mobility, competing work demands during clinic hours, and stigma [9], and community-based testing programs appear promising to improve linkage in these communities [9]. Interventions to improve linkage and retention may need to provide additional support and more patient-centered models of care to women, adolescents, and patients in rural and highly transitory communities.

Among patients who linked to care within 3 months, most (83.6%) linked within 1 week, suggesting that linkage support may be critical in the first weeks after patients test positive. Patients who linked to care within 1 week appeared to be slightly more likely to be retained in care, although this difference was not statistically significant. Similarly, in the US National Surveillance system, among 20,572 people diagnosed with HIV and linking within 3 months, 81.7% linked within the first month (linkage within 1 week was not assessed) and the percentage of patients achieving viral suppression was higher among patients who linked more quickly [31]. In addition, in a study of US adolescents, shorter time to linkage was associated with better engagement in care [32]. While it is likely that early linkage is indicative of greater patient motivation, these findings suggest that early supportive engagement with patients may be important to ensure that that they link to care.

The data from this study suggests that some facilities may be scheduling multi-month visits for newly initiated patients, rather than more frequent visits according to national guidelines. This observation should be assessed further, particularly in light of new efforts to promote differentiated models of care for different types of patients. At present, differentiated care usually is meant to begin after 6-12 months of ART. Also, the median time to first follow-up was 28 days which may indicate a change in regimens away from nevirapine-based regimens which require a lead-in dose; it could be dangerous for patients on these regimens to wait more than 2 weeks for an appointment. In the future, it will be important to better understand the factors driving the timing of appointments by clinicians and implications on patient retention. There is need to understand why nearly 15% of clients never returned for a second appointment. This could have important long-term implications as the national program rolls out its Test and Start strategy, especially if readiness for ART is not assessed appropriately before initiating ART.

This study is limited by its reliance on facility staff reporting and retrospective data from health records. Because data collection was limited to the 20 facilities in our study, we could not confirm outcomes of patients who were LTFU at the facility, meaning that some of the patients who appeared to be LTFU may in fact be in care elsewhere and our estimates of linkage and retention are likely to be underestimates. Nevertheless, our findings highlight relatively low levels of linkage and retention such that even if the percentages of patients LTFU are overestimates, they remain alarmingly high. Due to funding limitations, we were only able to follow up patients to assess retention after 6 months rather than 1 year or more as reported in some other studies [33]. Finally, because we chose to focus on poorly-performing facilities, the findings of this study may not be generalizable to sites with much lower attrition. Strengths of this study include the focus on struggling facilities to more readily identify gaps and areas for improvement and detailed contextual data from facility and district staff.

Beyond understanding patterns and predictors of linkage and retention, the next step will be designing and implementing effective interventions to improve engagement in care. Although the number and quality of studies is limited, several interventions have been identified that may be effective. Home-based HIV testing and counseling with follow-up support and community-based testing appear to be promising strategies to increase linkage [34, 35] and may work well in Uganda, [36, 37] although robust studies remain limited; an ongoing trial is assessing the impact of this type of program in rural Uganda [38]. A meta-analysis of interventions in sub-Saharan Africa to improve the rate or timing of ART initiation identified point-of-care CD4 testing, home based testing, improved clinic operations, home-based testing interventions, patient directed services, and HIV/TB integration as promising or effective [35]. A systematic review focused on adolescents, given high attrition in this group, identified education and counseling, financial incentives, increasing clinic accessibility, and adolescent-specific services as promising to improve linkage and retention [39]. Finally, a systematic review of studies to increase linkage, adherence, and retention noted key themes including poverty, food insecurity, transportation and housing constraints, and unmet mental health needs as factors limiting the success of public health HIV interventions [40]. To maximize the impact of health systems strengthening activities, all of these factors must be addressed.

Looking to the future, a number of new programs and policies may impact patient engagement in care. New Test and Start policies could reduce retention because asymptomatic patients will be put on treatment and may be less likely to stay in care due to the side effects they experience on treatment. However, better drug regimens that will soon be standard first line regimens such as Dolutegravir may play an important role in improving retention because of increased tolerability and reduced side effects [41–44]. Through these programmatic changes, focus on maintaining and/or improving patient linkage and retention will be essential. Health systems strengthening activities such as improved follow-up of patients through phone calls and home visits, enhanced counseling and education, and better facility oversight may be important to improve patient outcomes. However, while health systems strengthening activities may have a significant impact on patient engagement in care, factors outside of the facility will continue to limit the success of any facility-level interventions. Addressing all factors leading to low linkage, retention, and follow-up, including persistent stigma around HIV, will be crucial to reduce patient morbidity and mortality and eliminate HIV globally.

## Conclusion

This study suggests that patients who test HIV-positive may benefit from immediate support in order to link to care in a timely manner. There should be additional focus on adolescents to improve linkage to care and retention in care in this age group. Further investigation is needed on clinician appointment scheduling practices and implications on retention.

## Additional files

Additional file 1: Supplementary Results. This section provides additional facility-level information on existing barriers to patient follow-up, counseling, linkage to care, and retention in care. (DOCX 116 kb)

Additional file 2: **Table S1.** This table provides the percentage of individuals linked to care and retained in care according to different definitions of linkage and retention, as well as the median (IQR) days from HIV diagnosis to linkage among patients who did link to care. (DOCX 14 kb)

## Abbreviations

ART: Antiretroviral therapy
DHIS: District Health Information System
IQR: Interquartile range
LTFU: Lost to follow-up

## References

[1] UNAIDS (2015). HIV and AIDS estimates: Uganda.

[2] UNAIDS (2014). 90-90-90- an ambitious target to help end the AIDS epidemic.

[3] Uganda MoH (2014). Q3 2014 Uganda ART quarterly report.

[4] Koole O, Tsui S, Wabwire-Mangen F, Kwesigabo G, Menten J, Mulenga M, Auld A, Agolory S, Mukadi YD, Colebunders R (2014). Retention and risk factors for attrition among adults in antiretroviral treatment programmes in Tanzania, Uganda and Zambia. Tropical Med Int Health.

[5] Organization UMoHSACPatWH (2015). Implementing the test and treat policy for all HIV-infected children under 15 years of age: Uganda’s experience.

[6] USAID (2015). Strengthening Uganda's Systems for Treating AIDS Nationally (SUSTAIN): quarterly report January 1-March 31.

[7] O'Laughlin KN, Kasozi J, Rabideau DJ, Parker RA, Mulogo E, Faustin ZM, Greenwald KE, Doraiswamy S, Walensky RP, Bassett IV (2017). The cascade of HIV care among refugees and nationals in Nakivale Refugee Settlement in Uganda. HIV Med.

[8] Bogart LM, Naigino R, Maistrellis E, Wagner GJ, Musoke W, Mukasa B, Jumamil R, Wanyenze RK (2016). Barriers to linkage to HIV Care in Ugandan Fisherfolk Communities: a qualitative analysis. AIDS Behav.

[9] Bogart LM, Wagner GJ, Musoke W, Naigino R, Linnemayr S, Maistrellis E, Klein DJ, Jumamil RB, Mukasa B, Bassett IV (2017). A comparison of home-based versus outreach event-based community HIV testing in Ugandan Fisherfolk Communities. AIDS Behav.

[10] Mugasha C, Kigozi J, Kiragga A, Muganzi A, Sewankambo N, Coutinho A, Nakanjako D (2014). Intra-facility linkage of HIV-positive mothers and HIV-exposed babies into HIV chronic care: rural and urban experience in a resource limited setting. PLoS One.

[11] Geng EH, Odeny TA, Lyamuya R, Nakiwogga-Muwanga A, Diero L, Bwana M, Braitstein P, Somi G, Kambugu A, Bukusi E (2016). Retention in care and patient-reported reasons for undocumented transfer or stopping care among HIV-infected patients on antiretroviral therapy in Eastern Africa: application of a sampling-based approach. Clin Infect Dis.

[12] Namusobya J, Semitala FC, Amanyire G, Kabami J, Chamie G, Bogere J, Jain V, Clark TD, Charlebois E, Havlir DV (2013). High retention in care among HIV-infected patients entering care with CD4 levels >350 cells/muL under routine program conditions in Uganda. Clin Infect Dis.

[13] Asiimwe SB, Kanyesigye M, Bwana B, Okello S, Muyindike W (2016). Predictors of dropout from care among HIV-infected patients initiating antiretroviral therapy at a public sector HIV treatment clinic in sub-Saharan Africa. BMC Infect Dis.

[14] Okoboi S, Ding E, Persuad S, Wangisi J, Birungi J, Shurgold S, Kato D, Nyonyintono M, Egessa A, Bakanda C (2015). Community-based ART distribution system can effectively facilitate long-term program retention and low-rates of death and virologic failure in rural Uganda. AIDS Res Ther.

[15] World Health Organization (2015). Consolidated guidelines on HIV testing services.

[16] World Health Organization (2016). Consolidated guidelines on the use of antiretroviral drugs for treating and preventing HIV infection: recommendations for a public health approach.

[17] Kayigamba FR, Bakker MI, Fikse H, Mugisha V, Asiimwe A, Schim van der Loeff MF (2012). Patient enrolment into HIV care and treatment within 90 days of HIV diagnosis in eight Rwandan health facilities: a review of facility-based registers. PLoS One.

[18] Menon AA, Nganga-Good C, Martis M, Wicken C, Lobner K, Rothman RE, Hsieh YH (2016). Linkage-to-care methods and rates in U.S. emergency department-based HIV testing programs: a systematic literature review brief report. Acad Emerg Med.

[19] Miettinen O (1985). Theoretical epidemiology: principles of occurrence research.

[20] Bott S, Neuman M, Helleringer S, Desclaux A, Asmar KE, Obermeyer CM, Group MS (2015). Rewards and challenges of providing HIV testing and counselling services: health worker perspectives from Burkina Faso, Kenya and Uganda. Health Policy Plan.

[21] Shubber Z, Mills EJ, Nachega JB, Vreeman R, Freitas M, Bock P, Nsanzimana S, Penazzato M, Appolo T, Doherty M (2016). Patient-reported barriers to adherence to antiretroviral therapy: a systematic review and meta-analysis. PLoS Med.

[22] Bhoobun S, Jetty A, Koroma MA, Kamara MJ, Kabia M, Coulson R, Ansumana R, Jacobsen KH (2014). Facilitators and barriers related to voluntary counseling and testing for HIV among young adults in Bo, Sierra Leone. J Community Health.

[23] Bobat R, Archary M, Lawler M (2015). An update on the HIV treatment cascade in children and adolescents. Curr Opin HIV AIDS.

[24] Auld AF, Shiraishi RW, Mbofana F, Couto A, Fetogang EB, El-Halabi S, Lebelonyane R, Pilatwe PT, Hamunime N, Okello V (2015). Lower levels of antiretroviral therapy enrollment among men with HIV compared with women - 12 countries, 2002-2013. MMWR Morb Mortal Wkly Rep.

[25] Takuva S, Brown AE, Pillay Y, Delpech V, Puren AJ (2017). The continuum of HIV care in South Africa: implications for achieving the second and third UNAIDS 90-90-90 targets. AIDS.

[26] GARPR (WHO, UNAIDS, UNICEF) 6 July 2015; 76 reporting low- and middle-income countries, as referenced in fact sheet to the WHO consolidated guidelines on HIV testing services. 2015. http://apps.who.int/iris/bitstream/10665/179931/1/WHO_HIV_2015.20_eng.pdf.

[27] Vogt F, Tayler-Smith K, Bernasconi A, Makondo E, Taziwa F, Moyo B, Havazvidi L, Satyanarayana S, Manzi M, Khogali M (2015). Access to CD4 testing for rural HIV patients: findings from a cohort study in Zimbabwe. PLoS One.

[28] Gunn JK, Asaolu IO, Center KE, Gibson SJ, Wightman P, Ezeanolue EE, Ehiri JE (2016). Antenatal care and uptake of HIV testing among pregnant women in sub-Saharan Africa: a cross-sectional study. J Int AIDS Soc.

[29] Smolak A (2014). A meta-analysis and systematic review of HIV risk behavior among fishermen. AIDS Care.

[30] Opio A, Muyonga M, Mulumba N (2013). HIV infection in fishing communities of Lake Victoria Basin of Uganda--a cross-sectional sero-behavioral survey. PLoS One.

[31] Hall HI, Tang T, Johnson AS, Espinoza L, Harris N, McCray E (2016). Timing of linkage to care after HIV diagnosis and time to viral suppression. J Acquir Immune Defic Syndr.

[32] Philbin MM, Tanner AE, DuVal A, Ellen JM, Xu J, Kapogiannis B, Bethel J, Fortenberry JD, Adolescent Trials Network for HI (2016). HIV testing, care referral, and linkage to care intervals affect time to engagement in care for newly diagnosed HIV-infected adolescents in 15 adolescent medicine clinics in the United States. J Acquir Immune Defic Syndr.

[33] Fox MP, Rosen S (2015). Retention of adult patients on antiretroviral therapy in low- and middle-income countries: systematic review and meta-analysis 2008-2013. J Acquir Immune Defic Syndr.

[34] Ruzagira E, Baisley K, Kamali A, Biraro S, Grosskurth H, Working Group on Linkage to HIVC (2017). Linkage to HIV care after home-based HIV counselling and testing in sub-Saharan Africa: a systematic review. Tropical Med Int Health.

[35] Fox MP, Rosen S, Geldsetzer P, Barnighausen T, Negussie E, Beanland R (2016). Interventions to improve the rate or timing of initiation of antiretroviral therapy for HIV in sub-Saharan Africa: meta-analyses of effectiveness. J Int AIDS Soc.

[36] Ware NC, Wyatt MA, Asiimwe S, Turyamureeba B, Tumwesigye E, van Rooyen H, Barnabas RV, Celum CL (2016). How home HIV testing and counselling with follow-up support achieves high testing coverage and linkage to treatment and prevention: a qualitative analysis from Uganda. J Int AIDS Soc.

[37] Kyaddondo D, Wanyenze RK, Kinsman J, Hardon A (2012). Home-based HIV counseling and testing: client experiences and perceptions in Eastern Uganda. BMC Public Health.

[38] Kiene SM, Kalichman SC, Sileo KM, Menzies NA, Naigino R, Lin CD, Bateganya MH, Lule H, Wanyenze RK (2017). Efficacy of an enhanced linkage to HIV care intervention at improving linkage to HIV care and achieving viral suppression following home-based HIV testing in rural Uganda: study protocol for the Ekkubo/PATH cluster randomized controlled trial. BMC Infect Dis.

[39] MacPherson P, Munthali C, Ferguson J, Armstrong A, Kranzer K, Ferrand RA, Ross DA (2015). Service delivery interventions to improve adolescents' linkage, retention and adherence to antiretroviral therapy and HIV care. Tropical Med Int Health.

[40] Tucker JD, Tso LS, Hall B, Ma Q, Beanland R, Best J, Li H, Lackey M, Marley G, Rich ZC (2017). Enhancing public health HIV interventions: a qualitative meta-synthesis and systematic review of studies to improve linkage to care, adherence, and retention. EBioMedicine.

[41] Raffi F, Rachlis A, Brinson C, Arasteh K, Gorgolas M, Brennan C, Pappa K, Almond S, Granier C, Nichols WG (2015). Dolutegravir efficacy at 48 weeks in key subgroups of treatment-naive HIV-infected individuals in three randomized trials. AIDS.

[42] Raffi F, Rachlis A, Stellbrink HJ, Hardy WD, Torti C, Orkin C, Bloch M, Podzamczer D, Pokrovsky V, Pulido F (2013). Once-daily dolutegravir versus raltegravir in antiretroviral-naive adults with HIV-1 infection: 48 week results from the randomised, double-blind, non-inferiority SPRING-2 study. Lancet.

[43] Cahn P, Pozniak AL, Mingrone H, Shuldyakov A, Brites C, Andrade-Villanueva JF, Richmond G, Buendia CB, Fourie J, Ramgopal M (2013). Dolutegravir versus raltegravir in antiretroviral-experienced, integrase-inhibitor-naive adults with HIV: week 48 results from the randomised, double-blind, non-inferiority SAILING study. Lancet.

[44] Molina JM, Clotet B, van Lunzen J, Lazzarin A, Cavassini M, Henry K, Kulagin V, Givens N, de Oliveira CF, Brennan C (2015). Once-daily dolutegravir versus darunavir plus ritonavir for treatment-naive adults with HIV-1 infection (FLAMINGO): 96 week results from a randomised, open-label, phase 3b study. Lancet HIV.

