# Supplementary material for: Assessing linkage to and retention in care among HIV patients in Uganda and identifying opportunities for health systems strengthening: a descriptive study
Source: BMC Infect Dis. 2018 Mar 23;18:138. doi: 10.1186/s12879-018-3042-8 (PMC5865302; doi:10.1186/s12879-018-3042-8)
Supplement: Supplementary file 1 — Supplementary Results. This section provides additional facility-level information on existing barriers to patient follow-up, counseling, linkage to care, and retention in care. (DOCX 116 kb) [file 12879_2018_3042_MOESM1_ESM.docx]

**SUPPLEMENTARY RESULTS**

Additional facility-level qualitative and quantitative data was collected to better understand potential reasons that patient linkage and retention were low. Most facilities reported having systems in place for counseling and follow-up of patients. Specifically, most or all facilities noted having a cadre of staff to conduct patient follow-up and counseling (100%), using appointment books for patient recordkeeping (100%), contacting patients who missed appointments through telephone follow-ups (80%), contacting patients who could not be reached over the phone via home visits (70%), and offering adherence counseling (100%). However, a number of issues were identified with these systems. For example, for phone follow-ups to patients who missed appointments, facility staff noted often not being able to reach patients due to incorrect numbers or patients not having access to a personal phone line, lack of adequate airtime/equipment/finances, and logistical challenges due to power cuts and patchy phone networks. Similarly, issues identified with completing successful home visits to patients included being given the wrong address, long travel distances and limited accessibility of patient residences, limited funding for staff transport/logistical support, and stigma associated with visiting patients at their homes. Moreover, while counseling was happening in theory, many facility staff noted a lack of private space for counseling and inadequate staff training on patient counseling and education, making it difficult to complete quality training. Study staff noted that patient appointment books were inconsistently completed, making it difficult to discern whether follow-up was conducted for patients who missed appointments.

Other challenges identified at facilities further limited the ability of staff to provide quality HIV care for patients. Drug stockouts were relatively common; 65% of facilities reported experiencing stockouts of ARVs or cotrimoxazole in the previous year. Facility staff noted limited waiting and storage spaces, limiting efficiency. Multiple registers specific to HIV testing and treatment were in place across the facility, making it difficult to trace patients to assess which individuals were LTFU. Many facilities did not have a single unique number that was given to each patient across all services, making it challenging to directly link across registers, and no tracking system was in place to assess whether patients successfully linked to care. Many patients in the ART register were missing an ART care card (25%) and some facilities reported re-using patient care cards because they did not have sufficient cards for new patients. Transfer logs were not being used consistently to document patients transferring out of the facility and many appointment dates appeared to be missing. Facility staff noted patient-level challenges including stigma and sizeable transient populations of fisherfolk and farmers at some facilities. Finally, a less measureable but important potential contributor to program quality may have been poor supervision provided by facility in-charges/program managers as well as poor motivation of program staff; supervision and motivation appeared to vary greatly by facility.
