# Supplementary material for: Assessing linkage to and retention in care among HIV patients in Uganda and identifying opportunities for health systems strengthening: a descriptive study
Source: BMC Infect Dis. 2018 Mar 23;18:138. doi: 10.1186/s12879-018-3042-8 (PMC5865302; doi:10.1186/s12879-018-3042-8)
Supplement: Supplementary file 2 — Table S1. This table provides the percentage of individuals linked to care and retained in care according to different definitions of linkage and retention, as well as the median (IQR) days from HIV diagnosis to linkage among patients who did link to care. (DOCX 14 kb) [file 12879_2018_3042_MOESM2_ESM.docx]

**Table S1.** Patient linkage and retention patterns at 20 facilities in Uganda.

| **Measure** | **% / Median (IQR)** |
| --- | --- |
| **Linkage to care (N=928 HIV-positive patients)** |  |
| Linked to care on same day as HIV diagnosis | 29.1% |
| Linked within 1 week | 46.6% |
| Linked within 1 month | 53.0% |
| Linked within 3 months | 55.6% |
| Days from HIV diagnosis to linkage (among patients who linked) | 0 (0-6) |
| **Retention in care (N=678 patients newly initiated on ART)** |  |
| At least 6 appointments in 6 months | 6.9% |
| At least 1 appointment in the previous quarter (months 3-6) | 71.7% |
| At least 4 appointments in 6 months | 58.1% |
| Fully adherent^1^ to visit schedule | 45.1% |
| Came to first follow-up appointment | 85.5% |

^1^Defined as coming within 1 week of all scheduled appointments.
